# Modulation of Basophils' Degranulation and Allergy-Related Enzymes by Monomeric and Dimeric Naphthoquinones

**DOI:** 10.1371/journal.pone.0090122

**Published:** 2014-02-28

**Authors:** Brígida R. Pinho, Carla Sousa, Patrícia Valentão, Jorge M. A. Oliveira, Paula B. Andrade

**Affiliations:** 1 REQUIMTE/Laboratório de Farmacognosia, Departamento de Química, Faculdade de Farmácia, Universidade do Porto, Porto, Portugal; 2 REQUIMTE/Laboratório de Farmacologia, Departamento de Ciências do Medicamento, Faculdade de Farmácia, Universidade do Porto, Porto, Portugal; University Paris Sud, France

## Abstract

Allergic disorders are characterized by an abnormal immune response towards non-infectious substances, being associated with life quality reduction and potential life-threatening reactions. The increasing prevalence of allergic disorders demands for new and effective anti-allergic treatments. Here we test the anti-allergic potential of monomeric (juglone, menadione, naphthazarin, plumbagin) and dimeric (diospyrin and diosquinone) naphthoquinones. Inhibition of RBL-2H3 rat basophils' degranulation by naphthoquinones was assessed using two complementary stimuli: IgE/antigen and calcium ionophore A23187. Additionally, we tested for the inhibition of leukotrienes production in IgE/antigen-stimulated cells, and studied hyaluronidase and lipoxidase inhibition by naphthoquinones in cell-free assays. Naphthazarin (0.1 µM) decreased degranulation induced by IgE/antigen but not A23187, suggesting a mechanism upstream of the calcium increase, unlike diospyrin (10 µM) that reduced degranulation in A23187-stimulated cells. Naphthoquinones were weak hyaluronidase inhibitors, but all inhibited soybean lipoxidase with the most lipophilic diospyrin, diosquinone and menadione being the most potent, thus suggesting a mechanism of competition with natural lipophilic substrates. Menadione was the only naphthoquinone reducing leukotriene C_4_ production, with a maximal effect at 5 µM. This work expands the current knowledge on the biological properties of naphthoquinones, highlighting naphthazarin, diospyrin and menadione as potential lead compounds for structural modification in the process of improving and developing novel anti-allergic drugs.

## Introduction

Allergy is an abnormal immune response against non-infectious environmental substances, named allergens [1]. Allergy comprises chronic disorders associated with reduced quality of life, such as eczema or allergic rhinitis, and potential life-threatening reactions, including anaphylaxis and severe asthma episodes [2]. The prevalence of allergic disorders has been increasing globally, affecting roughly 25% of people in developed countries. This increased prevalence has been associated to environmental changes, such as air pollution and ambient temperature increment, which may induce early springs with increased airborne pollen [1]. On the other hand, the “hygiene hypothesis” suggests that reduced exposure to microorganisms in early life contributes to an immune system more susceptible to allergic and autoimmune diseases [3]. In the allergic process, immune cells, such as mastocytes, eosinophils, basophils and macrophages, release several mediators (including histamine and leukotrienes) that are responsible for allergic symptoms [4]. Additionally, these mediators may promote the development of different diseases, by inducing pathophysiological changes in the affected organs [1], [5]. A classic example is the role of leukotrienes in the pathogenesis of asthma and allergic rhinitis, by inducing bronchoconstriction and increased vascular permeability [6]. Thus, the increased allergy prevalence, together with the deleterious consequences of repetitive exposure to allergens, stresses the need for new strategies to induce immunological tolerance to allergens as well as new anti-allergic drugs [1].

Nature continues to be a rich source of novel bioactive molecules, and several plant extracts have been probed for anti-allergic properties. Namely, the grape seed extract of *Vitis vinifera* L. [7], the rhizomes extract of *Dioscorea membranacea* Pierre ex Prain & Burkill, in which the main active compound was a quinone (dioscoreanone) [8], or the leaf extract of *Rhinacanthus nasutus* Kuntze, which is rich in naphthoquinones [9]. Naphthoquinones are compounds constituted by two carbonyl groups in a naphthalene skeleton, naturally occurring in plants, fungi, bacteria and lichens, where they playing key survival roles, namely in defence against pathogens [10]. The high biological potential of naphthoquinones has been used in the search of new drugs, such as new anti-allergic drugs. In fact, 1,4-naphthoquinones isolated from *R. nasutus* were capable of inhibiting RBL-2H3 basophils' degranulation in the micromolar range, and decreasing tumour necrosis factor (TNF)-α and interleukin production [9]. Further studies, with synthetic naphthoquinones, support their anti-allergic properties: 2-alkyl/arylcarboxamido derivatives of 3-chloro-1,4-naphthoquinone inhibited the degranulation on mastocytes stimulated with compound 48/80 [11]. On the other hand, allergic reactions are common after temporary tattoos with henna (derived from *Lawsonia inermis* L.), where lawsone (2-hydroxy-1,4-naphthoquinone) is the main compound responsible for dye properties. Still, allergic reactions to henna have been attributed only to the occasional additive *p*-phenylenediamine [12], [13].

**Figure 1:**
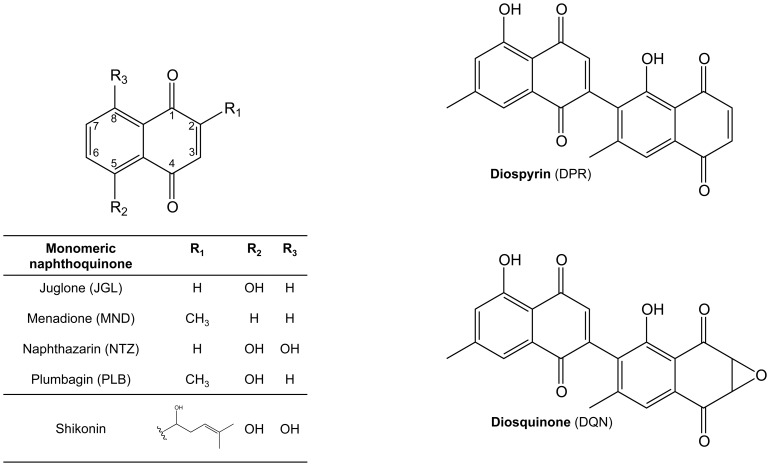
Chemical structures of monomeric and dimeric naphthoquinones.

In this work we studied the anti-allergic properties of naphthoquinones commonly produced by *Diospyros* species: diospyrin (DPR), diosquinone (DQN), juglone (JGL), menadione (MND), naphthazarin (NTZ) and plumbagin (PLB) (Fig. 1). Several biological activities have been attributed to these compounds, namely, anti-inflammatory [14], antitumor [15] and antimicrobial [16], but anti-allergic properties were only identified for menadione [17] and plumbagin [18]. To our knowledge, no anti-allergic data exists for the other *Diospyros*' naphthoquinones. For the initial screening, two different stimuli were used to induce RBL-2H3 basophils' degranulation (IgE/antigen or the calcium ionophore A23187) and the released *β*-hexosaminidase and histamine were quantified. Additionally, hyaluronidase and lipoxidase inhibition by naphthoquinones were evaluated, as well as the inhibition of leukotrienes production in IgE/antigen-exposed RBL-2H3 cells.

## Materials and Methods

### Test compounds

Plumbagin (PLB), naphthazarin (NTZ), menadione (MND) and juglone (JGL) were obtained from Sigma-Aldrich (St. Louis, MO, USA). Diospyrin (DPR) and diosquinone (DQN) (Fig. 1) were isolated from the root barks of *Diospyros chamaethamnus* Dinter ex. Mildbr. [19] and their purity was evaluated by HPLC-DAD as before [14].

### Chemicals and reagents

Medium, buffers and supplements for cell culture, including Earle's Balanced Salt Solution (EBSS) were from Gibco, Invitrogen™ (Grand Island, NY, USA) and bovine albumin fraction V solution 7.5% (BSA) was from Sigma-Aldrich (St. Louis, MO, USA).

Hyaluronic acid sodium salt from *Streptococcus equi*, hyaluronidase from bovine tests (type IV-S; EC 3.2.1.35), lipoxidase from *Glicine max* (L.) Merr. (type V-S; EC 1.13.11.12), as well as degranulation stimuli, monoclonal anti-dinitrophenyl (DNP) antibody produced in mouse, dinitrophenyl albumin (DNP-BSA) and calcium ionophore A23187 were from Sigma-Aldrich (St. Louis, MO, USA). Leukotriene C_4_ EIA kit was from Abcam (Cambridge, United Kingdom). All other chemicals were from Sigma-Aldrich (St. Louis, MO, USA), with the exception of 3-(4,5-dimethylthiazol-2-yl)-2,5-diphenyltetrazolium bromide (MTT), which was from Duchefa Biochemie (Haarlem, The Netherlands).

### Cell assays

Rat basophilic leukaemia cell line, RBL-2H3, was from the American Type Culture Collection (ATCC®) (LGC Standards S.L.U., Barcelona, Spain). Cells were cultured in DMEM (Dulbecco's Modified Eagle's Medium) + GlutaMAX™- I supplemented with 15% heat inactivated foetal bovine serum, 100 U/ml penicillin and 100 µg/ml streptomycin. Cells were maintained under 5% CO_2_, at 37°C, in humidified air.

**Figure 2:**
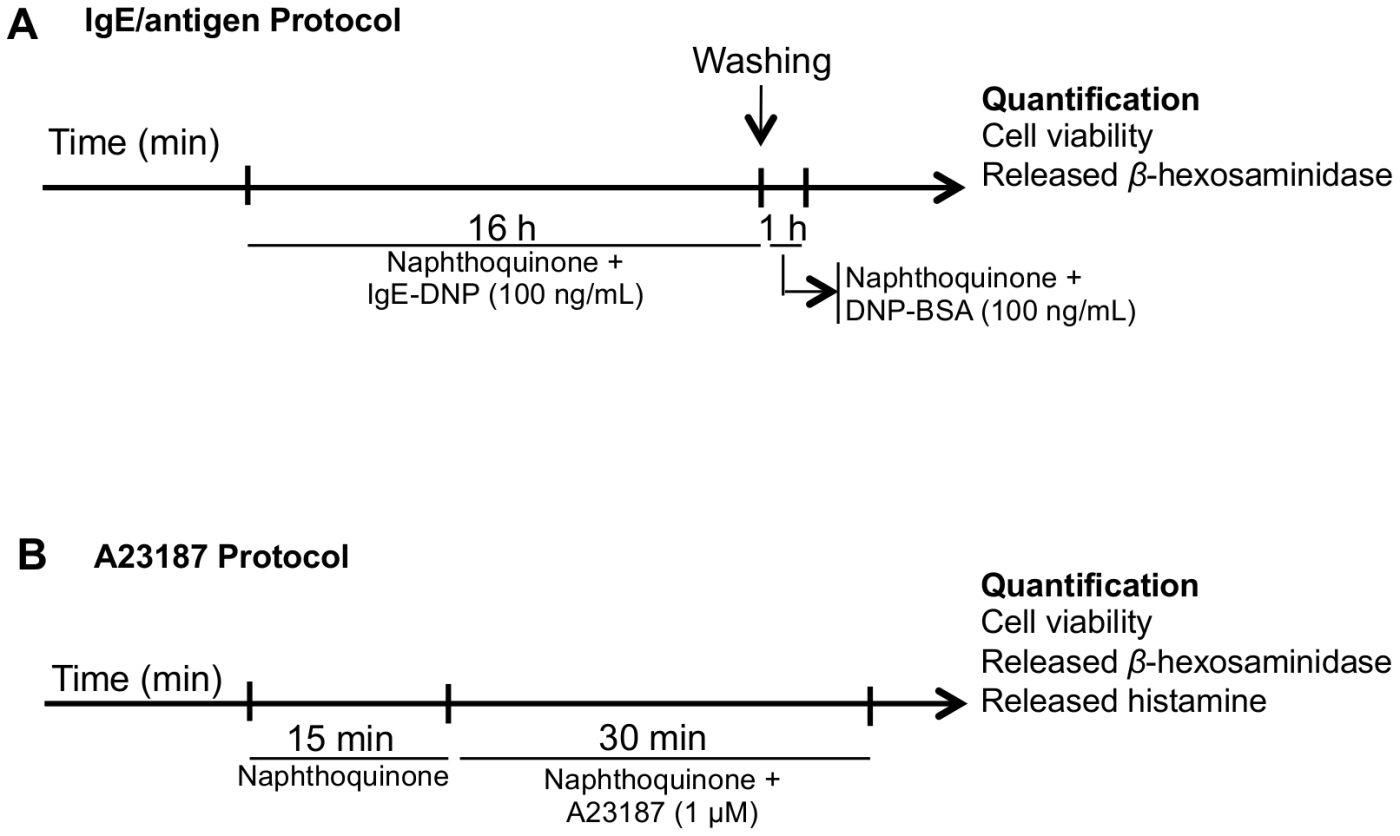
Schematic representation of protocols of RBL-2H3 basophils' degranulation assays using IgE/antigen (A) or calcium ionophore (A23187) (B) as stimuli.

RBL-2H3 cells were seeded at 3.0×10^5^ cells/mL in 24-wells plate (1 mL/well), and assayed after 24h at near-confluent stage (∼90%). Two different degranulation stimuli were used: calcium ionophore A23187 500 ng/mL (1 µM) and an immunologic stimulus (100 ng/mL IgE anti-DNP followed by 100 ng/mL DNP-BSA) that we refer to as IgE/antigen (Fig. 2). Both stimuli induced degranulation, which was quantified by *β*-hexosaminidase and histamine release. With IgE/antigen, *β*-hexosaminidase release (*p*-nitrophenolate absorbance) increased by 65.4±6.8% above basal (*n* = 16; *P*<0.001), whereas histamine increased from the basal value of 0.156±0.151 µM towards 0.521±0.186 µM (*n* = 10; *P*<0.05). With A23187, *β-*hexosaminidase release increased by 187±18.9% above basal (*n* = 12; *P*<0.001), whereas histamine increased towards 2.43±0.330 µM (*n* = 12; *P*<0.001). Thus, the ability of naphthoquinones to reduce *β*-hexosaminidase release was quantified for both stimuli, whereas effects upon histamine were only quantified with the A23187 stimulus, given the low histamine release and poor signal to noise achieved with IgE/antigen.

Quercetin was used as positive anti-degranulation control [20] and the anti-degranulation effects of diospyrin, diosquinone, juglone, menadione, naphthazarin and plumbagin (Fig. 1), were studied at non-toxic concentrations (determined by testing several concentrations of each compound and using the MTT assay to evaluate the effect on cell viability). The concentrations of the tested compounds in the degranulation assays with different stimuli were kept constant [0.1 µM (NTZ), 1 µM (DQN and PLB), 5 µM (MND) and 10 µM (JGL)], except for diospyrin and quercetin, where a 10 fold higher concentration was also tested in the A23187 assay.

**Figure 3:**
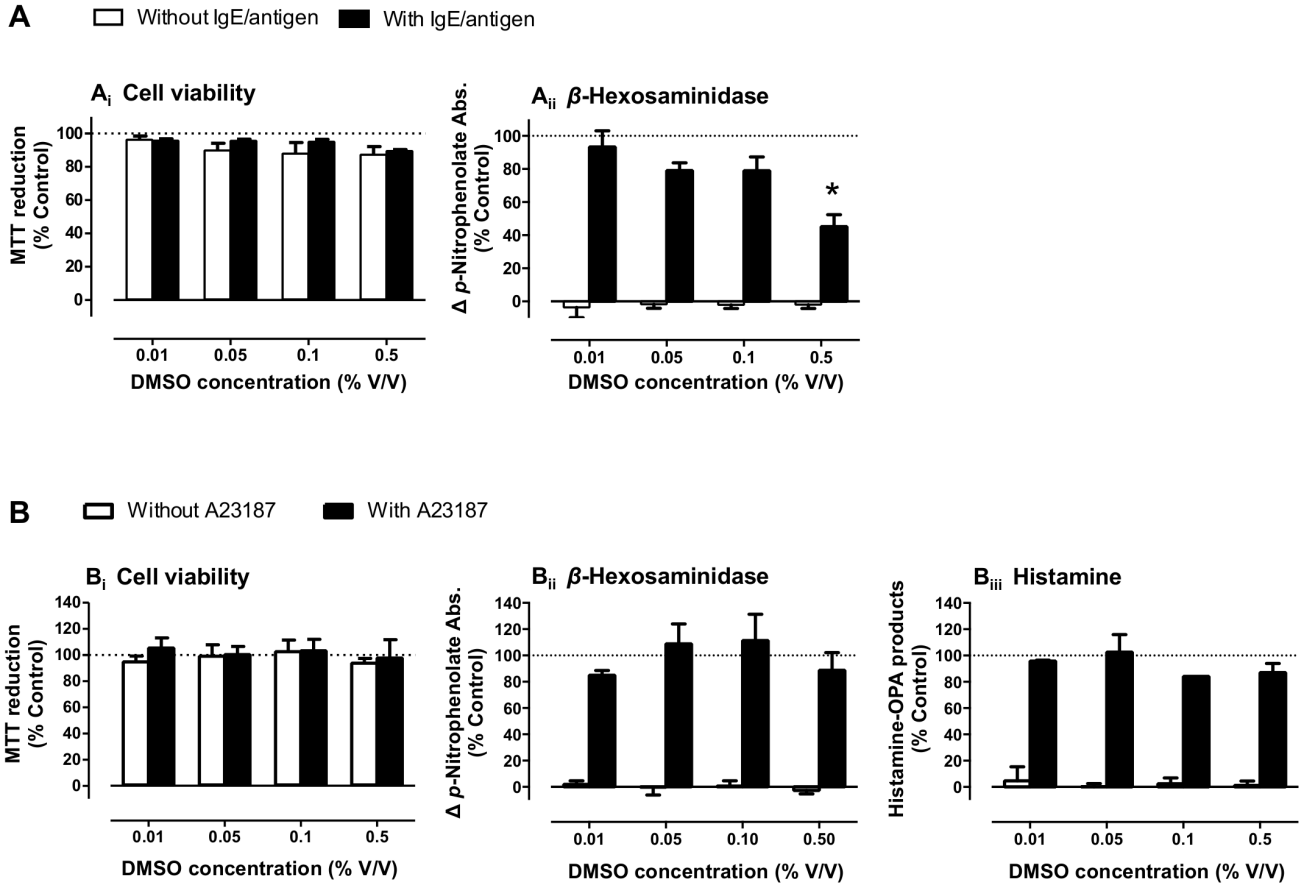
Solvent (DMSO) effect on RBL-2H3 basophils' degranulation assays. Effects of DMSO on cell viability (*i*), *β*-hexosaminidase (*ii*) and histamine release (*iii*) in cells stimulated with IgE/antigen (A) or with calcium ionophore (A23187) (B), *n* = 3–4. *P<0.05, One way ANOVA with Bonferroni post-hoc (*vs.* control).

A23187, quercetin and naphthoquinones stocks were dissolved in dimethyl sulfoxide (DMSO), aliquoted and stored at −20°C. We determined the maximal non-interfering solvent concentrations (Fig. 3; 0.1% and 0.5% DMSO for IgE/antigen and A23187 assays, respectively), as this was a limiting factor for testing higher naphthoquinone concentrations.

#### IgE/antigen assay

When the IgE/antigen was used as stimulus, cells were incubated during 16 h with 100 ng/ml IgE anti-DNP and with individual naphthoquinones diluted in culture medium. After washing twice with Dulbecco's phosphate buffered saline (DPBS), cells were treated for 1 h, at 37°C, with 100 ng/ml DNP-BSA and with individual naphthoquinones diluted in EBSS supplemented with 0.1% BSA (Fig. 2A) [21]. After treatments, supernatants were collected in order to quantify released *β*-hexosaminidase and released histamine, while cell viability assay was performed on adherent cells.

#### A23187 assay

Before treatment with A23187, cells were incubated with individual naphthoquinones during 15 min, at 37°C. After that, A23187 1 µM was added and cells incubated for 30 min, at 37°C (Fig. 2B). Compounds were freshly diluted prior to cell exposure using EBSS supplemented with 0.1% BSA [21]. *β*-hexosaminidase and histamine release was quantified in supernatants, whereas the MTT cell viability assay was performed on adherent cells.

#### Cell viability

Cell viability was assessed by the cellular dehydrogenases' dependent reduction of MTT to formazan, which was quantified by the measurement of optical density at 550 nm using a microplate reader (Multiskan ASCENT Thermo®), as described before [14].

#### Released *β*-hexosaminidase quantification

The release of *β*-hexosaminidase from stimulated-RBL-2H3 cells was measured as previously described [21]. In a 96-wells plate, 50 µl of substrate solution [*p*-nitrophenyl N-acetyl-D-glucosamine 1.3 mg/ml in citrate buffer (pH 4.5)] were added to 30 µl of supernatant. The plate was incubated at 37°C, during 1 h. The reaction was stopped by the addition of 80 µl of NaOH 0.5 M and the reaction product, *p*-nitrophenolate, was measured spectrophotometrically at 405 nm, in a microplate reader (Multiskan ASCENT Thermo®).

#### *β*-hexosaminidase inhibitory activity

Beyond avoiding *β*-hexosaminidase release, individual naphthoquinones may directly inhibit *β*-hexosaminidase enzymatic activity. For this, the inhibition of *β*-hexosaminidase enzymatic activity by naphthoquinones and quercetin was evaluated in an assay similar to the one described above: individual naphthoquinones (5 µl) were incubated with supernatant of degranulated cells where *β*-hexosaminidase is present (25 µl of supernatant of cells treated with A23187), in presence of 50 µl of substrate solution, during 1 h, at 37°C. The determination was made at 405 nm, in a microplate reader (Multiskan ASCENT Thermo®) [22].

#### Released histamine quantification

100 µl of NaOH 1 M and 25 µl of *o*-phthalaldehyde (OPA) 1% (w/v) were added to 500 µl of supernatant to convert histamine into fluorescent histamine-OPA-products. After 4 min incubation at room temperature, the reaction was stopped with of 50 µl of HCl 3 M. Precipitated proteins were removed by centrifugation at 14,000 rpm, during 3 min. The fluorescent histamine-OPA-products were quantified in the supernatant using 360 nm excitation and 450 nm emission in a microplate reader (Biotek Synergy HT®) [21]. Changes in histamine release are expressed as the difference between maximal and basal release, in percentage of control.

#### Leukotriene C_4_ quantification

Leukotriene C_4_ quantification was performed in the supernatant of IgE/antigen stimulated cells using a competitive enzyme immunoassay kit, according to the supplier's protocol (Abcam, Cambridge, United Kingdom) in a microplate reader (Multiskan ASCENT Thermo®) at 405 nm.

### Assays of enzymatic inhibition in cell-free systems

#### Hyaluronidase

The enzymatic reaction mixture was composed by 50 µl hyaluronic acid (5 mg/ml in water), 100 µl buffer pH 3.68 (HCOONa 0.2 M, NaCl 0.1 M and BSA 0.2 mg/mL), 200 µl water, 50 µl individual naphthoquinones solution and 50 µl hyaluronidase 600 U. The enzymatic reaction occurred during 1 h, at 37°C. The reaction product, *N*-acetyl-sugar, was quantified according Morgan-Elson colour reaction with minor modifications. The Morgan-Elson reaction was started by addition of 25 µl disodium tetraborate 0.8 M and subsequent heating in a boiling water bath during 3 min. After cooling, 750 µl *p*-dimethylaminobenzaldehyde (DMAB) was added and the reaction mixture was incubated at 37°C for 20 min. DMAB stock solution was prepared by dissolving 2 g DMAB in glacial acetic acid with 12.5% of HCl 10 N. This solution was further diluted in glacial acetic acid (1∶2) immediately before use. The measurement was made spectrophotometrically, at 560 nm, in a microplate reader (Multiskan ASCENT Thermo®) [23], [24]. Sodium cromoglycate was used as a positive control for inhibition [25]. DMSO was kept constant at 1%, without inducing significant enzyme inhibition.

#### Lipoxidase

Lipoxidase catalyses the oxidation of linoleic acid to the conjugated diene, 13-hydroperoxy linoleic acid, which was measured spectrophotometrically at 234 nm on a UV/visible spectrophotometer (UNICAM Helios α) [26]. The blank was measured in a reaction mixture with 20 µl of individual naphthoquinones solution, 1 ml of phosphate buffer (pH 9) and 20 µl of soybean lipoxidase 500 U. After 5 min pre-incubation at room temperature, the reaction was started by addition of 50 µl of substrate (linoleic acid) at 2 mM in ethanol. The reaction time was 3 min. DMSO was kept constant at 1.8%, without inducing enzyme inhibition. Quercetin was used as positive control [27], [28].

### Statistical analysis

Values are presented as mean±standard error of mean (SEM) of at least three independent experiments (*n*), performed at least in duplicate. Results concerning cell viability, released histamine and released *β*-hexosaminidase are expressed in percentage of the respective control. The normality of the values was evaluated by Shapiro-Wilk test, which was performed using Statistical Package for the Social Sciences version 20.0 (SPSS Inc., Chicago, IL, USA). Paired *t*-test or One-Way ANOVA with Bonferroni as post-hoc test were used, respectively, to compare two or more groups (GraphPad Prism version 5.0, San Diego, CA, USA). *P* values under 0.05 were considered statistically significant.

## Results

### Naphthazarin and diospyrin decreased RBL-2H3 degranulation

**Figure 4:**
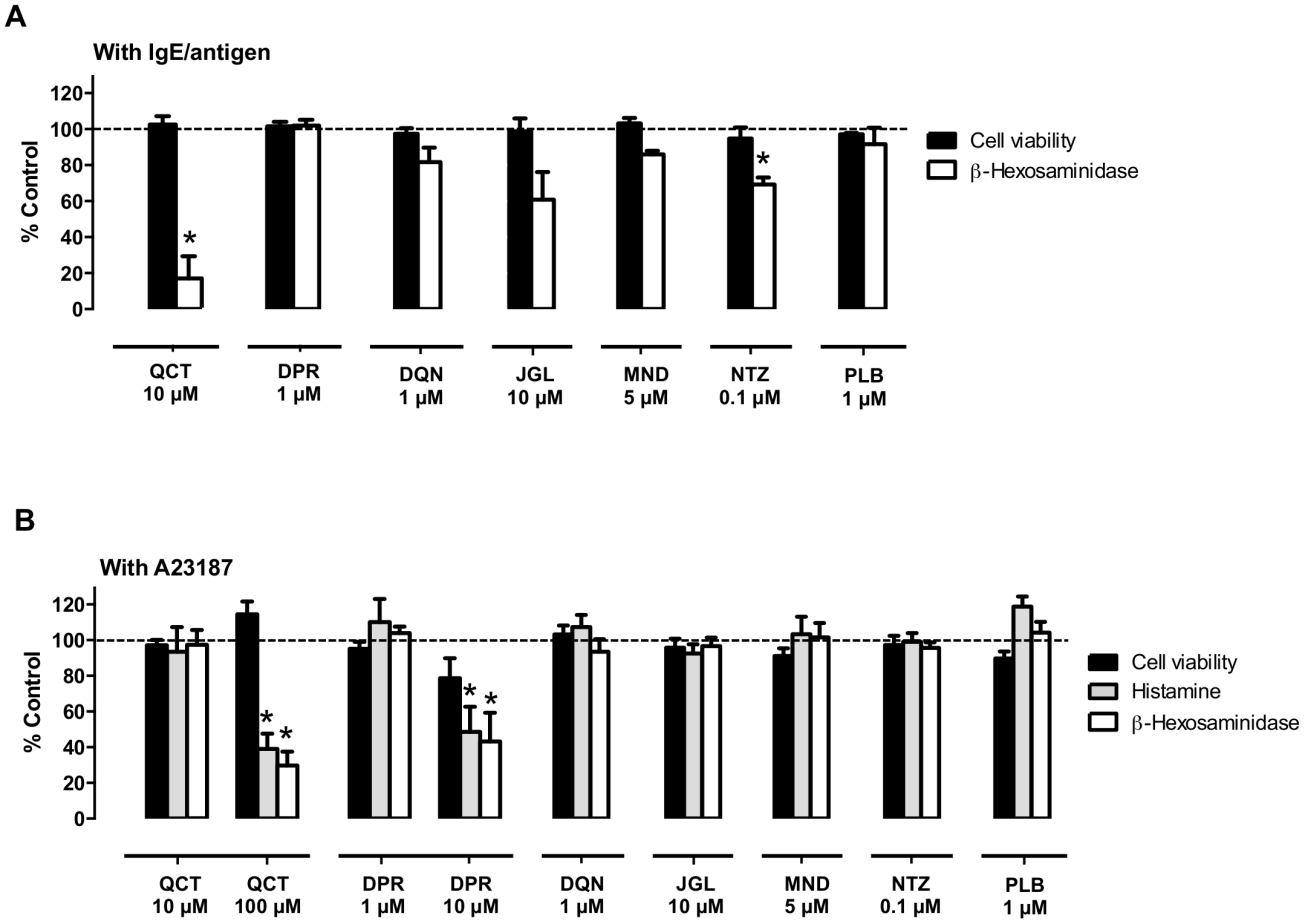
RBL-2H3 cells' degranulation inhibition by naphthoquinones. Effect on cell viability and *β*-hexosaminidase and histamine release in (A) IgE-antigen- or in (B) calcium ionophore (A23187)-stimulated cells treated with quercetin (QCT) and naphthoquinones [diospyrin (DPR), diosquinone (DQN), juglone (JGL), menadione (MND), naphthazarin (NTZ) and plumbagin (PLB)], *n* = 3–10. *P<0.05, paired *t* test to respective control (100 ng/mL IgE/DNP + 0.1% DMSO or 1 µM A23187 + 0.5% DMSO).

To test the anti-allergic properties of naphthoquinones, we evaluated their ability to inhibit RBL-2H3 basophils' degranulation evoked by two different stimuli: IgE/antigen (100 ng/mL, 16 h exposure) or the calcium ionophore A23187 (1 µM, 30 min exposure); Schematic protocols in Fig. 2. DMSO was used as a solvent, and we started by determining the maximal DMSO concentrations that could be used without interfering with the assays, and consequently the maximal concentrations that could be tested for the dissolved naphthoquinones (Fig. 3). DMSO at 0.5% decreased *β*-hexosaminidase release by 52.1±11.9% when IgE/antigen was used (*P*<0.05) (Fig. 3A), but it had no detectable effect on the release of *β*-hexosaminidase and histamine in A23187 stimulated cells (Fig. 3B), likely due to the shorter exposure time. Thus, the DMSO amount used in the IgE/antigen assay was 0.1%, while in the A23187 assay it was 0.5%, thus allowing for higher naphthoquinone concentration testing. None of the naphthoquinone concentrations used significantly affected cell viability, as assessed by the MTT reduction assay (Fig. 4; black bars).

In the IgE/antigen assay, naphthazarin (NTZ; 0.1 µM) was the only naphthoquinone able to significantly reduce degranulation, decreasing *β*-hexosaminidase release by 30.8±3.3% (*n* = 4; *P*<0.05) (Fig. 4A). Juglone (JGL; 10 µM) also displayed a tendency to reduce the degranulation induced by IgE/antigen, since it induced a decrease of 39.2±13.3% of released *β*-hexosaminidase (Fig. 4A).

In the A23187 assay, the positive control quercetin required a 10-fold higher concentration (100 µM) to reduce *β*-hexosaminidase release, when compared with the IgE/antigen assay. Diospirin (DPR) at the higher 10 µM concentration allowed by this assay, significantly decreased both *β*-hexosaminidase (56.8±14.6%) and histamine (51.4±12.8%) release, an amplitude of effect approaching that achieved with quercetin, and standing out amongst the other dimeric and monomeric naphthoquinones, none of which significantly affected degranulation at the maximal tested concentrations (Fig. 4B). None of the tested naphthoquinone concentrations induced degranulation in the absence of stimuli (IgE/Antigen or A23187) nor directly inhibited the *β*-hexosaminidase enzymatic activity (data not shown).

### Naphthoquinones are weak hyaluronidase inhibitors

**Figure 5:**
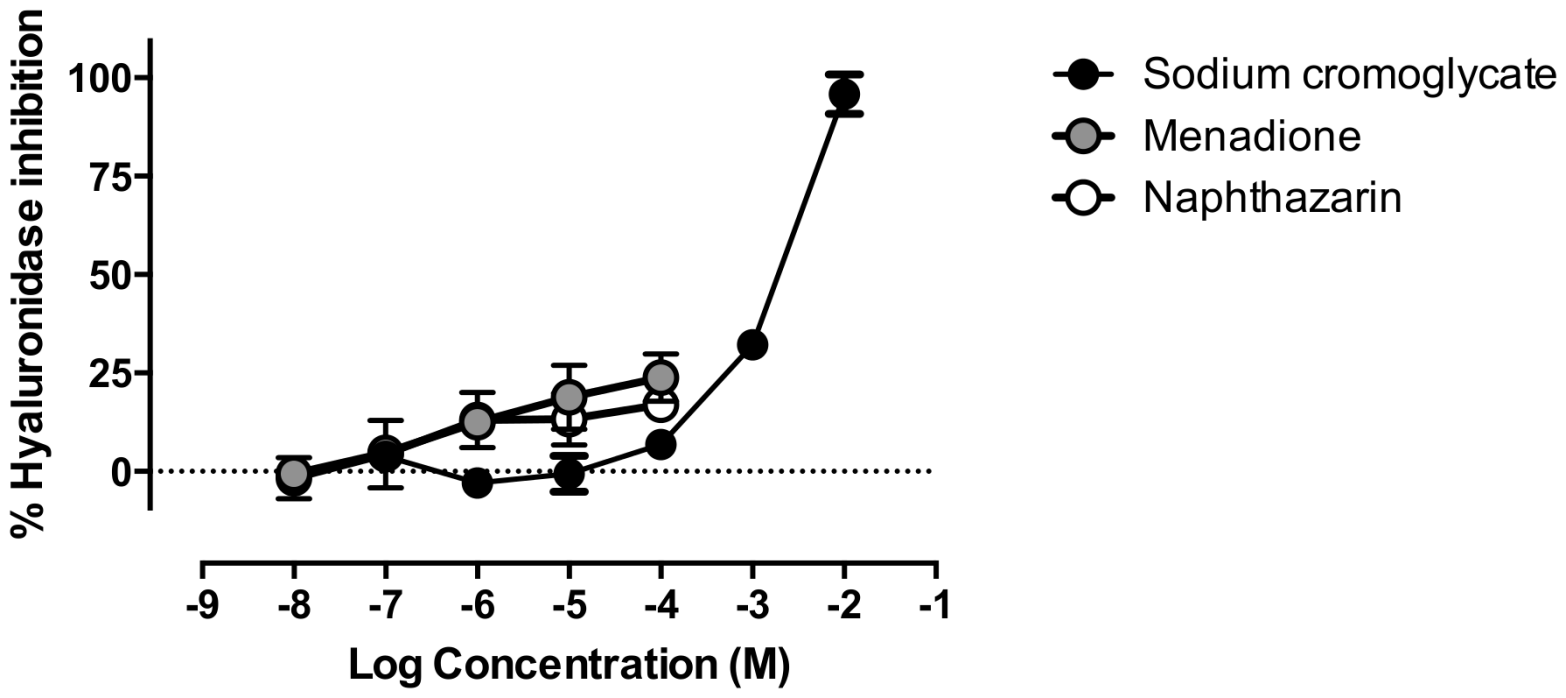
Concentration-dependent hyaluronidase inhibition by sodium cromoglycate (black circles) menadione (grey circles) and naphthazarin (white circles). *n* = 3–4.

Sodium cromoglycate was used as a positive control for hyaluronidase inhibition [25]. Complete hyaluronidase inhibition required 10 mM sodium cromoglycate (Fig. 5). DMSO amount precluded the testing of naphthoquinone concentrations above 100 µM. Only menadione and naphthazarin significantly inhibited hyaluronidase: at 100 µM, menadione and naphthazarin inhibited 23.8±6.06% and 16.9±2.45% the activity of the enzyme, respectively. Despite their modest inhibition of hyaluronidase, the level of inhibition induced by menadione and naphthazarin at 100 µM surpasses that achieved with the same sodium cromoglycate concentration (Fig. 5).

### Naphthoquinones effects on soybean lipoxidase and in leukotriene levels

**Figure 6:**
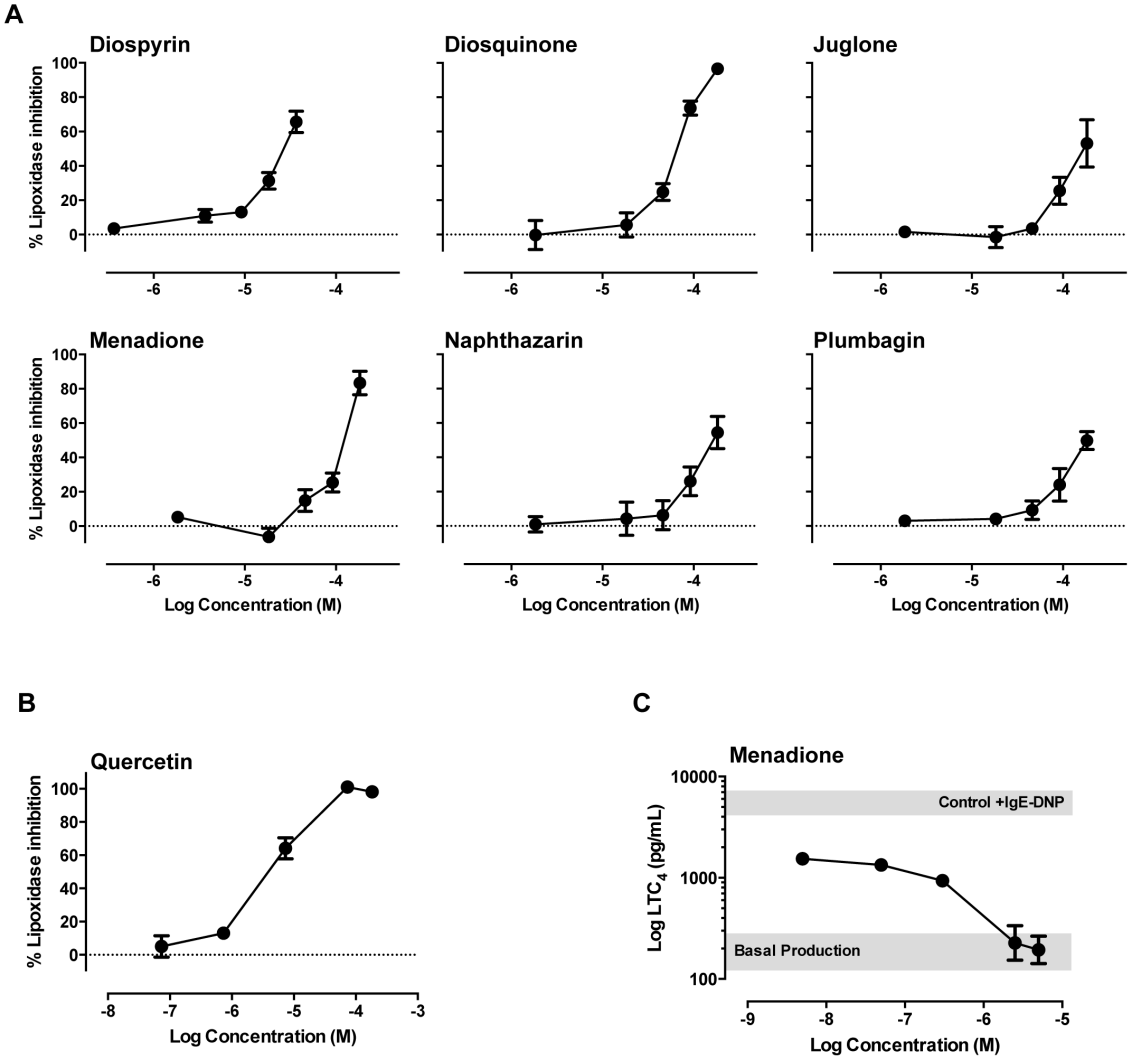
Lipoxidase inhibition and leukotriene C_4_ production. Soybean lipoxidase inhibition by individual naphthoquinones (A) and by quercetin (B) in a cell-free assay and inhibition of leukotriene C_4_ production in IgE/antigen-stimulated RBL-2H3 by menadione (C). Grey box represents LTC_4_ production of stimulated (upper) and non-stimulated (lower) control cells. *n* = 3–5.

All tested naphthoquinones and quercetin concentration-dependently inhibited soybean lipoxidase (Fig. 6A and 6B). Dimeric naphthoquinones, diospyrin and diosquinone, were the most potent with respective IC_50_ values of 28.9 and 83.8 µM, whereas all monomeric naphthoquinones displayed IC_50_ values above 100 µM, with the most potent being menadione with an IC_50_ of 128 µM (Table 1). These three most potent naphthoquinones were selected for an exploratory assay on leukotriene levels in IgE/antigen-stimulated RBL-2H3 cells. IgE/antigen treatment induced a robust increase in the levels of leukotriene (LT) C_4_ in the supernatant of RBL-2H3 cells, which was unaffected by either diospyrin (1 µM) or diosquinone (1 µM), but completely abrogated by menadione (5 µM). We thus performed a concentration response curve for menadione on LTC_4_ levels (Fig. 6C). Results showed that the lowest menadione concentration tested (5 nM) strongly decreased LTC_4_ levels evoked by IgE/antigen, suggesting interference with LTC_4_ synthesis mechanisms. Increasing menadione concentration-dependently decreased LTC_4_ levels until full inhibition of the IgE/antigen evoked release, with an IC_50_ of 0.34±0.018 µM (Fig. 6C).

**Table 1:** IC_50_ values (mean±SEM) for soybean lipoxidase inhibition by naphthoquinones and quercetin, using a cell-free assay.

**Compound**	**IC_50_ (µM)**
Quercetin	10.6±5.82
**Dimeric naphthoquinones**	
Diospyrin	28.9±2.53
Diosquinone	83.8±9.33
**Monomeric naphthoquinones**	
Juglone	>184
Menadione	128±9.38
Naphthazarin	142±27.1
Plumbagin	>184

## Discussion

**Figure 7:**
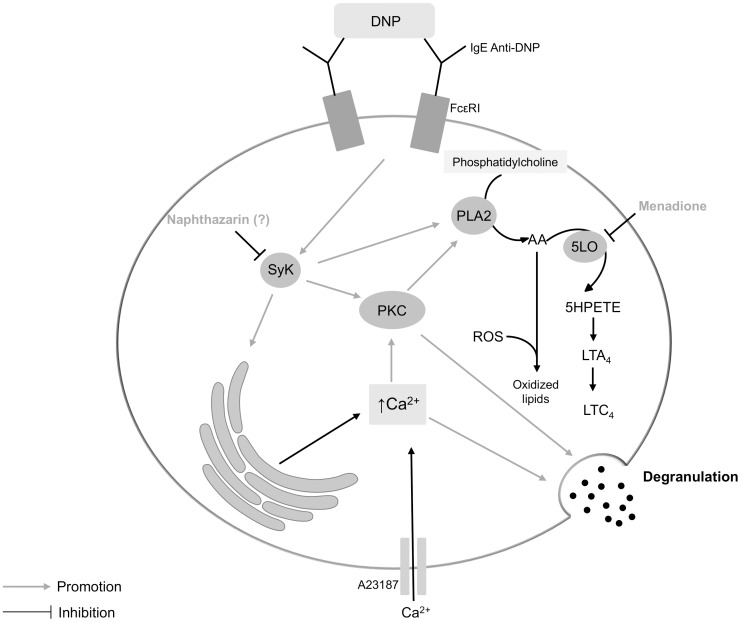
Simplified scheme of RBL-2H3 cells' degranulation pathways. The DNP antigen activates multiple signal transduction pathways via the IgE anti-DNP/FcεRI receptor complex. DNP receptor binding activates the immunoreceptor tyrosine activation motifs (ITAM)-Spleen tyrosine kinase (SyK) pathway that can be inhibited by shikonin [35] and probably by naphthazarin. Activated Syk catalyses protein phosphorylation of several proteins, leading indirectly to the activation of protein kinase C (PKC) that induces degranulation and the activation of phospholipase A2 (PLA2). PLA2 increases arachidonic acid (AA) bioavailability that can be converted in leukotrienes (LT) by 5-lipoxygenase (5LO; inhibited by menadione), or in oxidized lipids by means of ROS production. 5LO converts AA into 5-hydroperoxyeicosatetraenoic acid (5-HPETE), which is metabolised to an unstable epoxide, LTA_4_, and finally in LTC_4_, in RBL-2H3 cells. The increase in intracellular calcium by SyK pathway, as well as by A23187 promotes degranulation.

In this work we investigated the anti-allergic potential of monomeric and dimeric naphthoquinones (Fig. 1) by testing for inhibition of RBL-2H3 cells' degranulation. RBL-2H3 cells are a rat basophilic leukaemia cell line, expressing high affinity IgE receptors (FcεRI), being a model to study allergy and inflammation [29], [30]. Potent inflammatory mediators (histamine, proteases, cytokines, arachidonic acid metabolites and chemotactic factors) are released from immune cells after an allergic stimulus that can be IgE-dependent or IgE-independent [31]. To induce degranulation we used two previously described effective degranulation stimuli for RBL-2H3 [21]: IgE/antigen (simulation of IgE-dependent allergic response) and calcium ionophore (A23187; simulation of events that immediately precede degranulation: increase of intracellular calcium) (Fig. 7). These complementary stimuli assist characterization of the mechanisms by which the studied compounds reduce degranulation. In the present study, the release of immune cell degranulation markers – *β*-hexosaminidase and histamine [7] – was higher with ionophore than with IgE/antigen treatment, consistently with previous studies [32].

Naphthazarin (0.1 µM) decreased IgE/antigen-induced degranulation (Fig. 4A). However, the same naphthazarin concentration failed to reduce ionophore-induced degranulation (Fig. 4B), suggesting that naphthazarin's mechanism of degranulation inhibition is upstream of intracellular calcium increase. While this is the first report for naphthazarin, a similar mechanism was previously described for another naphthoquinone, namely, shikonin [33]. Shikonin reportedly inhibits the spleen tyrosine kinase (SyK), downstream from FcεRI activation, possibly explaining its anti-allergic properties [33]. Meaningfully, naphthazarin displays the highest structural similarities to shikonin, among the naphthoquinones in the present study. In fact, both compounds share a 5,8-dihydroxy-1,4-naphthoquinone core (Fig. 1). Thus, we propose that naphthazarin and shikonin act through a similar mechanism and that both C5 and C8 hydroxyls modulate direct enzyme interaction via hydrogen bonds [34]. However, other mechanisms of action, that need clarification, could explain the degranulation inhibition verified in presence of naphthazarin, such as a potential binding of naphthazarin to FcεRI or to IgE.

Diospyrin (10 µM) reduced degranulation in calcium ionophore-stimulated cells (Fig. 4B). Another naphthoquinone, acetylshikonin, was reported to attenuate ionophore-mediated intracellular calcium elevation in rat neutrophils [35]. While, attenuation of calcium elevation might partly explain the effects of both diospyrin and acetylshikonin, their spectrum of activity does not necessarily overlap, since acetylshikonin is reported to decrease leukotriene B4 and tromboxane A2 [35], whereas in the present study diospyrin was unable to reduce leukotrien C4, albeit in different cell models and stimuli. As diospyrin only reduced the A23187-induced degranulation, other common mechanisms of action beyond attenuation of intracellular calcium increase could be excluded, because if diospyrin acted at a level after the intracellular calcium increase, such as the SNARE (soluble N-ethylmaleimide-sensitive fusion factor attachment protein receptor) complex formation, diospyrin should have also inhibited the degranulation induced by IgE/antigen complex. Nevertheless, the range of tested concentrations was different.

Plumbagin was previously reported to exhibit anti-allergic properties, namely, at 5 µM plumbagin inhibited cytokines production by phytohemagglutinin-stimulated peripheral blood mononuclear cells (PBMC) [18]. In the present study, the maximum non-toxic concentration of plumbagin that could be tested in RBL-2H3 cells was 1 µM, and at that concentration plumbagin did not prevent degranulation evoked by either IgE/antigen or A23187.

Naphthazarin and diospyrin, shown capable of inhibiting RBL-2H3 basophils' degranulation in the present study, warrant further investigation in models such as primary mast cells. Several questions about the membrane permeability of naphthoquinones may be drawn; however, the effects of both naphthazarin (the most hydrophilic), and diospyrin (the most lipophilic) on RBL-2H3 degranulation strongly suggest that naphthoquinones can cross cell membranes. Menadione in particular is well known for its mitochondrial effects [36], and that requires crossing the plasma membrane. Also atovaquone, a monomeric naphthoquinone has oral bioavailability for the treatment and prevention of malaria in humans [37].

Following degranulation studies, we addressed the inhibition of enzymes involved in allergic responses: hyaluronidase and lipoxidase. Hyaluronidase increases vascular permeability in inflammation, by cleavage of internal *β*-N-acetyl-D-glucosaminidic linkages of hyaluronic acid [38], thus being a possible target for anti-allergic drugs. Our data shows that the tested monomeric and dimeric naphthoquinones are poor hyaluronidase inhibitors, with only menadione and naphthazarin displaying modest inhibitory activity (Fig. 5). 5-Lipoxygenase is a rate-limiting enzyme for leukotriene synthesis, converting arachidonic acid into 5-hydroperoxyeicosatetraenoic acid (5-HPETE). 5-HPETE is metabolised to an unstable epoxide LTA_4_, which is transformed to LTB_4_ or LTC_4_ according with the cell type and the enzymes present (Fig. 7) [39]. In RBL-2H3 cells, LTC_4_ is the major leukotriene released, while LTD_4_ and LTE_4_ are not produced [40]–[42]. Soybean lipoxidase is often used to model human 5-, 12- and 15-lipoxygenases, given the high catalytic domain similarity between plant and mammalian lipoxygenases [43]. All tested naphthoquinones exhibited lipoxidase inhibiting activity (Fig. 6A). Dimeric naphthoquinones (diospyrin and diosquinone), and the monomeric menadione were the most potent, showing the lowest IC_50_ values (Table 1). Considering that these three naphthoquinones are the most lipophilic of the studied compounds, our results raise the hypothesis that naphthoquinones inhibit lipoxidase by competing with natural lipophilic substrates.

Considering the results obtained with soybean lipoxidase, we tested the inhibition of leukotriene production by diospyrin, diosquinone and menadione. Menadione was the only naphthoquinone able to reduce leukotriene production, achieving full inhibition at 5 µM (Fig. 6C). Higher menadione concentrations (50–200 µM) were previously reported to reduce leukotriene production by inhibiting 5-lipoxygenase translocation to the nuclear membrane [17]. Given that menadione is a known oxidative stress generator [44], and that reactive oxygen species (ROS) may react with arachidonic acid forming oxidized lipids (Fig. 7) [45], decreased LTC_4_ with our lower menadione concentrations may stem from decreased arachidonic acid availability. Consitently with our concentration range, menadione was reported to inhibit prostaglandin H_2_ (PGH_2_) synthase via ROS production with an IC_50_ of 5 µM [46].

Concluding, we evaluated the anti-allergic properties of monomeric and dimeric naphthoquinones by studying the inhibition of RBL-2H3 basophils' degranulation and LTC_4_ production induced by allergic stimuli, as well as by the evaluation of inhibition of enzymes involved in allergic responses (main findings for each compound are summarized in Table 2). To our knowledge, this is the first study addressing the anti-allergic potential of diospyrin, diosquinone, naphthazarin and juglone. Naphthazarin and diospyrin reduced degranulation by different mechanisms of action. Naphthazarin and diospyrin acted, respectively, upstream and downstream of the intracellular calcium increase. In spite of being poor inhibitors of hyaluronidase, naphthoquinones inhibited lipoxidase and menadione reduced leukotriene production. Thus, this work expands the current knowledge on the biological properties of naphthoquinones, highlighting naphthazarin, diospyrin and menadione as potential lead compounds for structural modification in the process of improving and developing novel anti-allergic drugs.

**Table 2:** Maximal inhibition (mean±SEM) of the main studied targets and respective naphthoquinones' concentration.

Naphthoquinone	Degranulation inhibition (%)		Hyaluronidase inhibition (%)	Lipoxidase inhibition (%)
	IgE/antigen	A23187		
Diospyrin	< 15 (1 µM)	56.8±14.6 (10 µM)	< 15 (20 µM)	65.6±6.24 (36.7 µM)
Diosquinone	18.4±8.07 (1 µM)	< 15 (1 µM)	< 15 (100 µM)	96.5±0.92 (183.5 µM)
Juglone	39.2±13.3 (10 µM)	< 15 (10 µM)	< 15 (100 µM)	53.1±13.8 (183.5 µM)
Menadione	< 15 (5 µM)	< 15 (5 µM)	23.8±6.06 (100 µM)	83.4±6.80 (183.5 µM)
Naphthazarin	30.8±3.82 (0.1 µM)	< 15 (0.1 µM)	16.9±2.45 (100 µM)	54.4±9.33 (183.5 µM)
Plumbagin	< 15 (1 µM)	< 15 (1 µM)	< 15 (100 µM)	49.8±5.23 (183.5 µM)
